# A single amino acid substitution alter antigenicity of Glycosylated protein 4 of HP-PRRSV

**DOI:** 10.1186/s12985-016-0586-3

**Published:** 2016-07-25

**Authors:** Xinglong Wang, Zhenbin Wang, Hongyu Xu, Xiang Biao, Zengqi Yang

**Affiliations:** 1 College of Veterinary Medicine, Northwest A&F University, Yangling, Shaanxi China; 2 Northwest A&F University, No. 3 Taicheng Road, 712100 Yangling, Shaanxi China

## Abstract

**Background:**

Porcine reproductive and respiratory syndrome (PRRS) is an important pig endemic disease in pork-producing countries worldwide. The etiology, porcine reproductive and respiratory syndrome virus (PRRSV), is characterized by fast antigen variability. Glycosylated protein 4 (GP4) is a minor protein in PRRSV virion, but contributes to induce protective immune responses. However, the antigenic characterization of PRRSV GP4 and the role of the mutations in this protein in PRRSV evolution are not clear.

**Methods:**

Peptides chip scanning and peptide based ELISA was used to analyze the antigenic characterization of HP-PRRSV GP4. A total of 142 peptides printed on a chip were used to reveal the antigen reaction characteristics of the HP-PRRSV. The reactions of these peptides with HP-PRRSV-specific pig serum were scanned and quantified using the software PepSlide® Analyzer by fluorescence intensity. The active reaction regions (AR) were identified based on the scanning results and then the amino acids (aa) sequences of AR(s) is aligned among PRRSV strains for further identify the key aa site(s) impact the antigenicity of the protein. Peptide based ELISA is then reacted with PRRSV positive sera derived from pig inoculated with different PRRSV strains for further analysis the role of specific amino acid in AR.

**Results:**

The intensity plot was used to show the reactions of the peptides with PRRSV serum and it showed that enormously different response happened to various parts of GP4. The highest reaction intensity value reached 6401.5 against one peptide with the sequence DIKTNTTAASDFVVL. An AR from S29 to G56 was identified. Sequence alignment revealed various mutations in site 43 and possibly played an important role in this AR. Peptides ELISA reaction with sera from pigs inoculated with different PRRSV strain revealed that the change of aa in site 43 reduced the reaction of the peptide with PRRSV positive sera derived from pigs inoculated with the peptide related PRRSV strains.

**Conclusion:**

In this study, one AR covering S29 to G56 was identified in GP4. The aa in site 43 play an important role in determining the antigenic character of GP4. The continual mutations (S → G → D → N) occurred in this site alter the antigenicity of PRRSV GP4.

**Electronic supplementary material:**

The online version of this article (doi:10.1186/s12985-016-0586-3) contains supplementary material, which is available to authorized users.

## Background

Porcine reproductive and respiratory syndrome (PRRS) is an important pig disease that is endemic in pork-producing countries worldwide. The etiology of the disease is porcine reproductive and respiratory syndrome virus (PRRSV). This virus belongs to the recently approved family *Arteriviridae* [1, 2]. Other members of this family include equine arteritis virus, simian haemorrhagic fever virus and lactate dehydrogenase-elevating virus [1]. In most cases, PRRSV infection on piglets causes respiratory disorders with serious pneumonia and is responsible for high mortality [3]. By contrast, pregnant sows infected by the virus experience serious reproductive disorders, such as abortion, infertility, mummified foetuses and stillborn piglets [4].

PRRSV is fast evolving agent and its variations are widespread [5, 6] with significantly different in terms of pathogenicity [7]. An important incident in PRRSV evolution is the appearance of the highly pathogenic PRRSV (HP-PRRSV) in China [8]. The variants of this virus have gained many new characteristics, such as gene deletion in non-structure protein 2 (NSP2) [9], genetic variation in full genome, significantly enhanced pathogenicity and resistance to typical PRRSV-induced immune responses [6, 8]. Numerous studies have focused on elucidating the rules of virus variation to understand the mechanism of immune escape of the virus [10, 11]. Determining this mechanism is crucial in developing methods to control HP-PRRSV.

Glycosylated protein 4 (GP4) is a minor protein in PRRSV virion; it is presented in low quantities on the surface of the virion [12]. Nevertheless, GP4 has important functions in generating infectious PRRSV [13]. GP4, GP3 and unglycosylated 2b protein form a heterotetrameric complex in infected cells [13] and a further research proved that GP2a interacting with GP3, GP4 and GP5 are another member of the heterotetrameric complex [14]. The formation of such a complex is required to transport these proteins from the endoplasmic reticulum to the Golgi apparatus in infected cells prior to virion assembly. GP4 and GP2a proteins also specifically interact with the CD163 molecule, which is a receptor of PRRSV attachment [14].

GP4 contributes to the induction of protective immune responses identified in previous research [15, 16]. GP4-specific neutralizing antibodies were recognised as driving forces in PRRSV evolution [17]. Amino acid (aa) substitutions in the GP4-neutralizing epitope can abrogate antibody recognition, and these neutralizing antibodies might be responsible for the selection of neutralizing antibody-resistant variants with aa substitutions in the neutralizing epitope on GP4. Our previous research revealed that the fusing protein GP3–GP4–GP5 induces protective immune responses in mice [18]. The immune responses induced by GP3–GP4–GP5 are significantly stronger than those induced by GP3–GP5. Protective immune responses can also be observed in animals vaccinated by DNA harbouring GP4 [19, 20] and the recombinant protein GP4 expressed by baculovirus [21]. In the present study, peptide microarray and peptide based ELISA using 14 overlapping peptides (15 aa in length) was conducted to analyse the immune recognition rule of HP-PRRSV GP4.

## Methods

### Virus and sera

The GP4 amino acids sequence of the HP-PRRSV strain SY0608 [8] was used as the template. The hyper-immune SY0608-specific serum was provided by Dr. P. Jiang (Nanjing Agricultural University, China). The serum was collected from pigs immunised with killed vaccine derived from SY0608 and then challenged with the virulent strain. Secondary antibody from goat anti-swine IgG (H + L) DyLight680 was provided by PEPperPRINT GmbH. CH-1a and/or HuN4 infected pigs sera were given by Dr. Zijun Li (Northwest A&F University, China).

### GP4 peptides chip and peptides scanning

A total of 142 peptides with a 15 aa-long overlapping covered the full length of GP4 (without a signal peptide, from 23 to 178 aa). The first 15 peptides (CKPCFSSSLSDIKTN) were printed on the chip with 14 duplicated aa from the second, and by the analogy, the second peptide was duplicated 14 aa with third. Each peptide had two replicates on the chip. Surrounding the target peptide points were the interval flag peptides DYKDDDDKGG and HA peptides YPYDVPDYAG served used as controls.

The peptide chip was blocked with Rockland blocking buffer MB-070 for 60 min prior to the first assay. Pre-staining of the peptide array was performed by using the secondary goat anti-swine IgG (H + L) DyLight680 antibody at a dilution of 1:5000. It was used to investigate background interactions that could interfere with the subsequent main assays. Subsequent incubation of the peptide array with HP-PRRSV SY0608-specific serum at a dilution of 1:1000 in incubation buffer (PBS, pH 7.4 with 0.05 % Tween 20 and 10 % Rockland blocking buffer) was followed by staining with the secondary goat anti-swine IgG (H + L) DyLight680 antibody, which was then read by scanning intensities of 5 and 7 (red). Quantification of spot intensities and peptide annotations was performed using PepSlide® Analyzer.

A software algorithm broke down fluorescence intensities of each spot into raw foreground and background signals. This algorithm was used to calculate the standard deviation of the foreground median intensities. Averaged spot intensities for pre-staining with the secondary antibody and the main assay with the pig serum against the GP4 sequence from the N-terminus to the C-terminus were plotted to visualize the overall spot intensities and signal-to-noise ratios. Fluorescence intensity plots were created with OriginLab Origin V8.0 software.

### GP4 and GP4 antigen region analysis

The GP4 amino sequence of the HP-PRRSV strain SY0608 was blasted with the American type II PRRSV strain VR2332, MLV(GenBank: AF066183.4), the Chinese type II PRRSV strain CH-1a and the HP-PRRSV-like PRRSV strains Jsyx and QY2010. The software Megalin in DNAstar was used to align the sequence. Residues that differed from VR2332 were listed, while the consistency aa were represented by dots.

Lines with yellow or red colour were used to highlight the strong reaction peptides with PRRSV positive serum in GP4. The peptides with fluorescence intensity values between 1000 and 3000 were marked with pale yellow lines, whereas those with fluorescence intensity values higher than 3000 were marked with red lines.

One antigen reaction activity region (AR) from S29 to G56 was identified in GP4 on the basis of the peptide scan results and GP4 sequence analysis. To further analyse this AR, 68 strains of type II PRRSV GP3 AR S^29^ to G^56^ sequence were downloaded from GenBank. The access numbers are given in Table 1. The alignment was conducted in the software MEGA 6 [22], and some special sites were further marked using Microsoft PowerPoint.

**Table 1:** Reference strains from GenBank

Strain name	Access number	Strain	Access number	Strain name	Access number
CH-1a_	AY032626	CG2006	EU864231	CWZ-1-F3_	FJ889130
VR2332	AY150564	BJ_2007	EU825723	WUH1_	EU187484
CH2003	EU880440	GD_2007	EU109503	BJPG_	FJ950746
CH2002_	EU880438	07QN	FJ394029	LN	EU109502
HB-1	AY150312	HUB2	EF112446	HEB1_	EF112447
HB-2	AY262352	07NM	FJ393456	07HEN	FJ393457
CH2004	EU880439	GD2007	EU880433	BJSD_	FJ950747
PRRSV02	FJ175688	GDBY1	GQ374442	GS2008	EU880431
P*RRSV03*	FJ175689	GDQJ_	GQ374441	SHH_2007	EU106888
BJ-4_1996	AF331831	CBB-1-F3	FJ889129	BJsy06	EU097707
GS2002	EU880441	Henan-1_	EU200962	NM1	EU860249
GS2004	EU880443	HPBEDV_	EU236259	SX2007	EU880434
S1	DQ459471	07BJ	FJ393459	SX-09	HQ843181
PRRSV01	FJ175687	HUB1_	EF075945	SY0608	EU144079
QY2010	JQ743666	HuN	EF517962	TJ_2006	EU860248
CC-1_2006	EF153486	Jiangxi-3	EU200961	TP_2006	EU864233
SHB_2005	EU864232	Jsyx	EU939312	XH-GD_2007	EU624117
Em2007	EU262603	JX143	EU708726	XL2008	EU880436
SD-CXA_2008	GQ359108	JX2006	EU880432	YN2008	EU880435
APRRS_2009	GQ330474	JXA1	EF112445	HN1	AY457635
NB_04	FJ536165	JXwn06	EF641008	HN2007	EU880437
SX-1_2011	GQ857656	NX06	EU097706	Lelystad	M96262

**Table 2:** Synthesized peptides used in peptide based ELLISA in this study

Name	Sequence (N → C)	purity	Notes
VD-203	PVYITITANVTDENY	>75 %	Positive Control
VD-204	DIKTNTTAASDFVVL	>75 %	Hun4
VD-205	DIKTNTTAASGFVVL	>75 %	CH-1A
VD-206	DIKTNTTAASNFVVL	>75 %	Qy2010
VD-207	DIKTNTTAASSFVVL	>75 %	VR2332
VD-208	DIKTNTTAAASFAVL	>75 %	VR2332
VD-209	SPTIRKISQCRTAIG	>75 %	Negative Control

### Peptide ELSIA

A serials of fifteen  aa length peptides (Table 2) were designed and synthesized by company (DgPeptidesCo., Ltd, Hangzhou, China). The peptides were coded on the plates and stander PRRSV positive and negative sera from commercial ELISA kit (IDEXX) were used for optimizing the coding concentration.

Peptides were dissolved to 1 mg/ml following the instruction given by the company. And serial diluted peptides and PRRSV positive sera were used to select the optimal coating concentration. Briefly, Peptides in coating buffer (10 mM NaHCO_3_ buffer, pH 9.6) were coded on the flat bottom 96-well amino immobilizer plates (NUNC, USA) at 4 °C overnight. The coded plates were washed 5 times with PBST and were blocked with 200 μL blocking buffer (1 % BSA in PBST) at 37 °C for 2 h, and after 5 times washing, the plates were dried by patting on towel and stocked in -20 °C before using.

In reaction steps, the plates were thawed to room temperature and incubated with 100 μL of antisera in blocking buffer at 37 °C for 45 min. After 5 times washing, antibody binding was detected by incubation with 100 μL of pig specific HRP conjugates (Boster Biological Technology, Wuhan, China) diluted 1:1000 in blocking buffer at 37 °C for 45 min. The chromogen development was mediated by the addition of 100 μL freshly prepared substrate solution (3, 3′, 5, 5′-tetramethylbenzidine, Sigma). The reaction was stopped by addition of 50 μL of 4 M sulfuric acid and the OD was measured at 450 nm.

### Statistical analysis

Student *t*-test were used to analysis the different reactions of the peptides with different sera. Differences were considered to be statistically significant when the *p* ≤ 0.05.

## Results

### Peptide scan

**Fig. 1:**
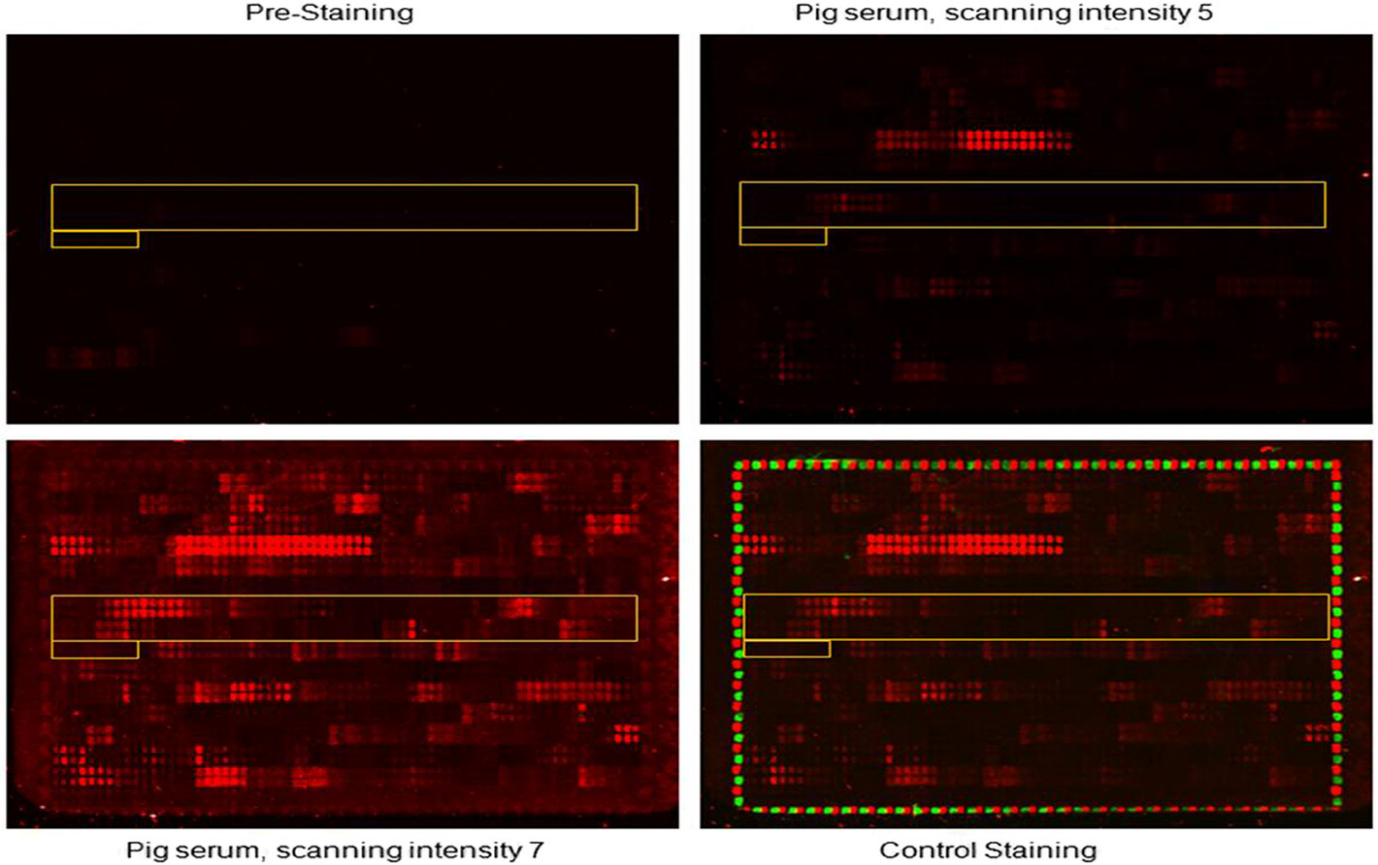
Images of peptide microarray. The 15 aa peptide printed on chip with a 14 aa duplicate. Each peptide has two replicates on the chip. GP4 peptide region was highlighted with yellow boxes. After being blocked, pre-staining was done with secondary goat anti-swine IgG (H + L) DyLight680 antibody. Subsequent incubation of peptide array with PRRSV SY0608 specific serum was followed by staining with secondary goat anti-swine and read-out at scanning intensities of 5 and 7 (red). HA and FLAG control peptides framing the peptide microarray were subsequently stained at high intensity

The reaction of peptide chip with antibody was read at scanning intensities of 5 and 7. Red fluorescence intensity reflects the strength of the reactions (Fig. 1) and green fluorescence (HA and Flag control) reports an equilibrium reaction of the peptides with antibodies in each region of chip (Fig. 1). The raw foreground and background signals read by machine are provided in Additional file 1 (“Mapping Raw Data” tab) and also the calculated standard deviation of foreground median intensities (Additional file 1 Mapping Summary” tab).

**Fig. 2:**
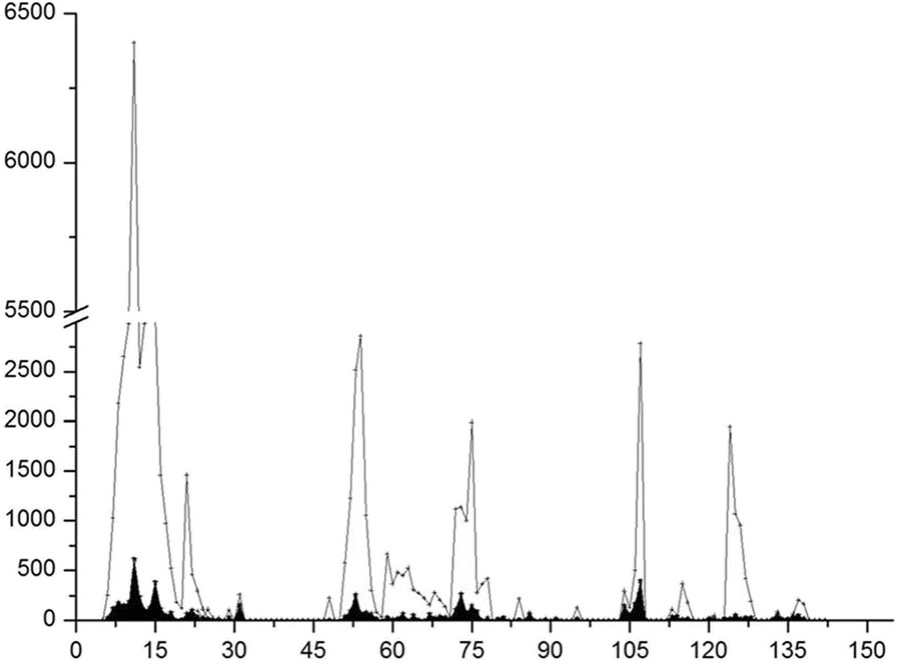
Peptides reaction intensity plots. Black area reflects pre-staining reaction results and Grey line indicates main assay reaction. In accordance with microarray scan and intensity map, strong and polyclonal response of pig serum was observed with overlapping peptides within the region from aa 29 to 56 in GP4. Relatively low intensities were found in pre-staining, though several sites show low affinity reaction with secondary antibodies

To visualize the overall spot fluorescence intensities, the averaged spot fluorescence intensity of pre-staining with secondary antibody was plotted in Fig. 2 (black shadow region). And also the main assay results with the pig HP-PRRSV specific serum against the GP4 sequence from the N-terminus to the C-terminus (Fig. 2, showed with gray line).

**Fig. 3:**
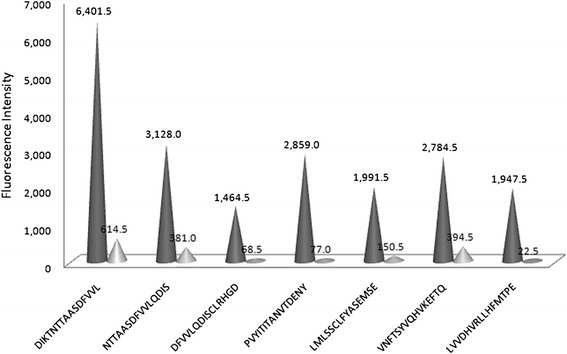
Epitope candidates in GP4. A total of 6 epitope candidates were elected from the peptides with high value of reaction (FI >1000) with PRRSV positive pig serum. Only one peptide was selected in one antigenic region

The fluorescence intensity plot (Fig. 2) was correlated with peptide and intensity maps (Additional file 2: “Peptide Map and Additional file 1: Intensity Map” tab). As showing in the Peptide Map and Intensity Map, a total of 22 peptides exhibited high fluorescence affinity with the polyclone antibodies and showed strong spot fluorescence intensities (>1000). The peptide DIKTNTTAASDFVVL showed the highest intensity at 6401.5. Six epitope candidates were identified because they showed relatively higher response than their neighbours with HP-PRRSV serum. Fig. 3 shows these peptides with their reaction values. Meanwhile, 69 peptides showed low response to the serum, and the intensity was recorded between 1 and 949. By contrast, 51 peptides showed no reaction.

**Fig. 4:**
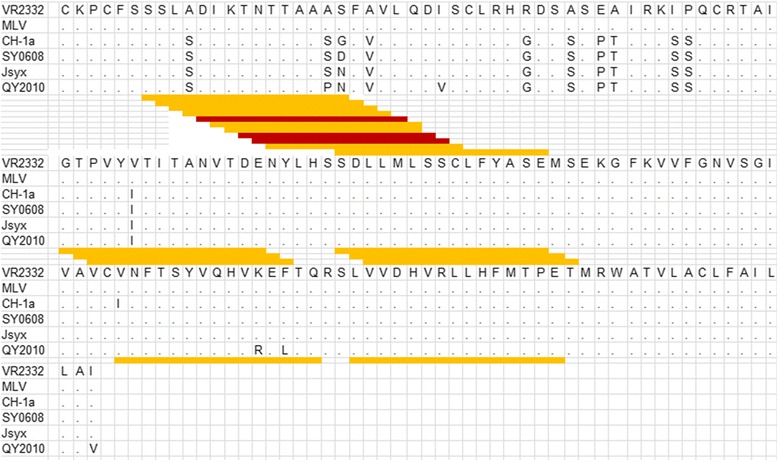
Multiple sequence alignment of GP4 amino acids sequence of SY0608, CH-1a, VR2332, MLV, Jsyx and YQ2010. Residues that differ fromVR2332 were listed, while the same aa used were represented as a dot. Antigenic regions identified by peptide scan were mark with yellow or red lines. Peptides with reaction fluorescence intensity value higher than 1,000 and lower than 3,000 were highlighted with yellow lines below the sequences. Reaction values higher than 3,000 were indicated with red lines

A map was generated on the basis of the averaged foreground median intensities and sequence alignment of SY0608, CH-1a, VR2332, MLV, Jsyx and YQ2010. The peptides with fluorescence intensity values between 1000 and 3000 were marked with yellow lines, whereas those with fluorescence intensity values higher than 3000 were marked with red lines (Fig. 4). Reaction AR was identified using the constructed map. Peptides from SSLSDIKTNTTAASD to SDFVVLQDISCLRHG showed relatively higher spot fluorescence intensities, and the region from S29 to G56 was the antigen reaction active region. Further analysis was performed on the AR to reveal the rule of HP-PRRSV variation.

### GP4 antigen region analyses

The aa sequence of GP4 of SY0608 (23-178 aa, without signal peptide) was aligned with CH-1a, VR2332, MLV, Jsyx and QY2010 (Fig. 4). Two aa mutations in 43 and 129 aa sites were found in SY0608 when compared with CH-1a, the suppositional ancestor of HP-PRRSV. Eleven amino acids (32, 42,43 45, 56, 59, 61, 62, 66, 67, 79 aa sites) were different from VR2332 or MLV, the typical type II PRRSV strains, in GP4 of SY0608, and the variants were relatively concentrated from 31 to 79 aa sites. Two identified ARs are in this region from 31 to 79 aa. New G → N mutations were observed in PRRSV isolates isolated around 2010, such as Jsyx and QY2010.

**Fig. 5:**
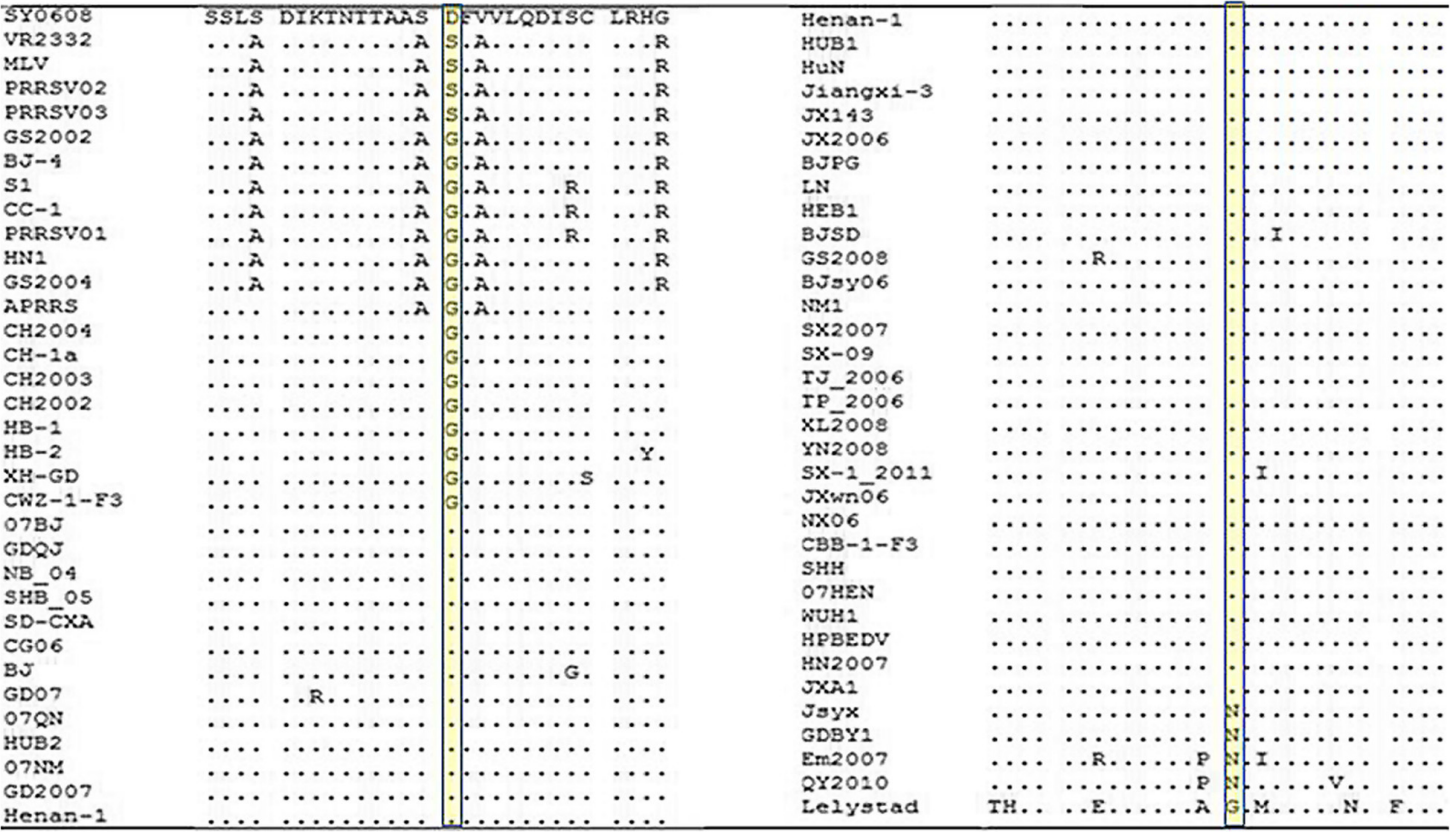
Multiple sequence alignment results of GP4 AR. Sixty-eight strains of AR (from 29 to 56 AAs of GP4) from reported PRRSV stains were used in this analysis. The mutation at site 43 in the AR was conspicuous. Continual mutation occurred at site 43 (S → G → D → N)

To deep look variation of GP4 in AR (from S29 to G56), a total of 68 ARs from different strains of PRRSV with complete genome in GenBank were downloaded and analyzed using MEGA 6 software [22]. The mutation in site 43 in GP4 was conspicuous (Fig. 5). Continual mutations (S → G → D → N, presented according to the appearance time of the related PRRSV strains) were observed in this site, but the other sites in this AR are relatively conserved. D → N mutation happened in isolates Jxyx, GDBY1, EM2007 and QY2010 in site 43 appeared more recently (Fig. 5).

### A further analysis of the mutation in the sites 43 in GP4 by peptide ELISA

**Fig. 6:**
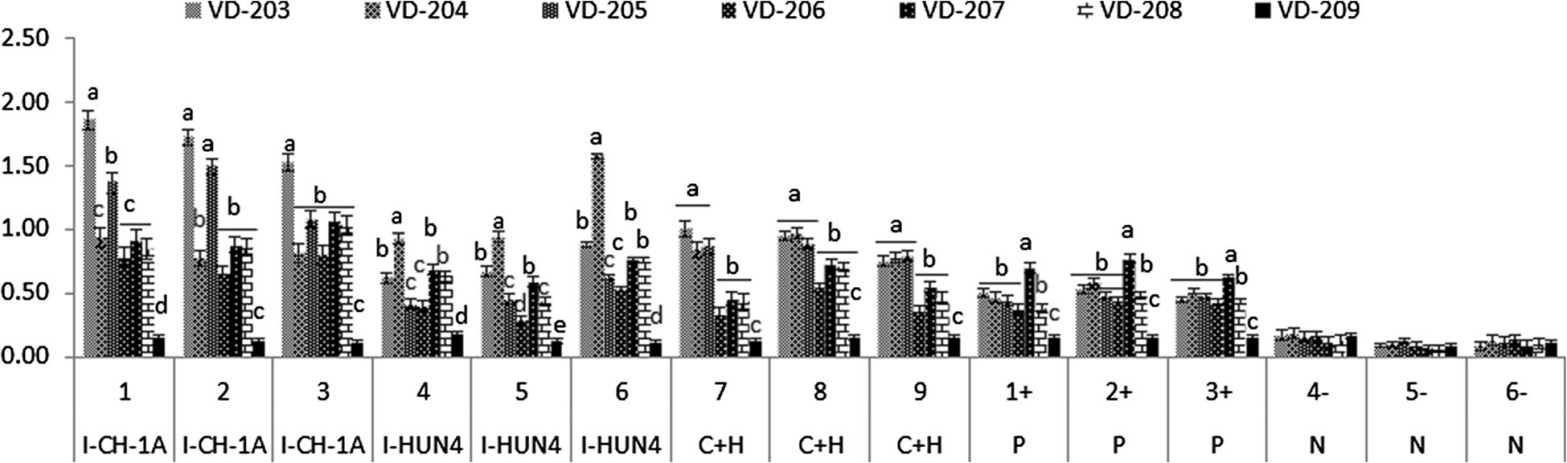
Peptide ELISA results. Nine peptides named as VD-203-VD209 were coded on the plates with concentration 2ug/ml. Nine PRRSV positive sera from pigs with clear virus exposure history (I-CH-1A from pigs infected with PRRSV stain CH-1a, I-HuN4 from pigs infected with PRRSV stain HuN4 and C + H infected with these two strains), 3 standard positive sera (P1+, P2+ and P3+) and 3 standard negative sera (N4-, N5- and N6-) were reacted with the peptides. Each reaction has three well repeats and the reaction differences were show by the bars. Significant different were considered when *p* < 0.05

After optimization, the concentration of 2ug/ml was selected according to serious dilution reaction results of the peptides and sera (data not shown). Three PRRSV positive (P1+, P2+ and P3+) and negative sera (N4-, N5- and N6-) from three different lots of the ELISA kit (IDEXX) were tested with the peptides ELISA. As shown in Fig. 6, all the OD 450 values of the negative sera were less than 0.2, which is acceptable according to the request for the negative control of the kit. The OD 450 value of all the positive sera reacted with each peptides were higher than 0.17, the cut-off value (Cut-off value = Mean + 3 Standard Deviation (SD) values of 30 known negative serum samples, selected by IDEXX ELISA kit) except peptide 209, which was the negative control.

The reactions of the peptides VD-203, VD-204 and VD-209 with sera from pigs infected with HuN4 indicate a good correlation between the peptides ELISA and peptides scanning (*R* = 0.9751, Data showed in supplementary).

As we found sites 43 in GP4 were important in determining antigen characters of the protein in conducting antigenic region analysis above, peptides VD-204- VD-208 derived from different PRRSV strains were synthesized. Nine PRRSV positive sera from pigs inoculated with CH-1a, HuN4 or both were used to react with peptides. As show in Fig. 6, peptides VD-204 and VD-205 show higher response values with the sea7, 8 and 9 (from pigs inoculated with both CH-1a and HuN4) when compared with that reacted with peptide VD-206-VD208, but the reactions to the sera between the VD-204 and VD-205 were not significant different. These provide basic information for the strain specific response in this peptide. What more, When analysis the reactions with CH-1a (sera 1-3) or HuN4 (sera 4-6) specific sera, significant difference were observed between the peptide VD-204 and VD-205. As only one aa difference was in VD-204 and VD-205, these data prove the important role of the aa sit 43.

To find out whether the mutations in sites 42 and 45 changed the antigenic character of HP-PRRSV, we synthesized peptides VD-207 and VD-208 with mutation in site 42 and 45, respectively. However, the reaction of the sea against peptides 207 and 208 were similar (Fig. 6), these revealed that the mutations in sites 42 and 45 in GP4 didn’t change the antigenic characters of the protein.

## Discussion

PRRSV is classified into two genotypes: I (European) and II (North American). These two genotypes share approximately 60 % genome sequence homology [23–25]. However, they have similar gene structure and cause almost same clinical diseases. In this study, the active antigenic region of HP-PRRSV, which belongs to type II PRRSV, was identified with peptide chip scanning method. The results showed that the immune recognition mostly occurred in the region between S29 and G56 in type II PRRSV. In contrast, the most active recognized region was in sites 57 to 68 as a neutralizing epitope was already identified in type I PRRSV in a previous research [16]. This neutralizing epitope was strain specific and quickly changed under the influence of vaccine immune pressure or the immune response induced by the virus infection [17]. However, peptides that covered this region showed no reaction with the PRRSV positive serum in our research. Therefore, GP4 is believed to be genotype specific in inducing linear epitope B cell response.

Polygenic analysis has been widely used to analyze gene variation in viruses [26, 27]. However, gene variation can occur in any parts. In many cases, the significant mutation cannot be determined by basing only on polygenic analysis result because many mutations may be non-meaning. HP-PRRSV share around 90 % homology with typical type II PRRSV, and gene mutation occurs in any parts in genome, both in structural and non-structural protein coding genes. HP-PRRSV and typical American PRRSV share 89.6 % homology in GP4, and different aa are relatively concentrated in sites 56 to 67. This variation region has been reported in type I PRRSV and may demonstrate an important function in driving virus variation. However, the results of this study revealed that this region was not recognized by linear epitope recognizing B cells.

Very interesting variation rules were determined in the analysis-identified antigenic region. The most striking mutation occurred in the aa in site 43. A series of mutations occurred in this site (S → G → D → N). D was widely used in HP-PRRSV, and G → D mutation was completed before the time when HP-PRRSV appeared, as “D” appeared in virus strains NB_04 and SHB_05, which were isolated in 2004 and 2005, respectively. But these two strains were not HP-PRRSV-like PRRSV, because no gene deletion occurred in their NSP2. In contrast, CH-1a, recognized as the ancestor of HP-PRRSV-like PRRSV [28], used a “G” in this site. Only two different amino acids were used in CH-1a and SY0608 (G43 → D; I129 → V), and V was used both in typical type II PRRSV and HP-PRRSV-like PRRSV. So “G → D” variation was recognized as a significant change. Considering that this change happened before the appearance of HP-PRRSV-like PRRSV, GP4 variation may occur in earlier stage during a variant PRRSV form. Based on this rule, a new character PRRSV strain already appeared as a new change in this site (D43 → N) has been found in PRRSV isolates (Jxyx, GDBY1, EM2007 and QY2010). In peptide based ELISA test, we found the different react role of the peptides with PRRSV strain specific sea. Strongest reactions were observed in the related peptide and the strain specific sea. Slacking down reaction was found whenever an amino acid change happening in this sits. In addition, the sits 42 and 45 in GP4 were less related with the antigenicity of the protein. Though more test are needed with more different PRRSV strains derived sera, we identified that the aa in site 43 plays an important role in determining the antigenicity of HP-PRRSV GP4. The mutation in this site weakens the reaction of HP-PRRSV derived peptides with antibody against classical type II PRRSV strain. This mutation may related with the immune evasion ability gained by HP-PRRSV, but further researches to confirm the epitope is a neutralizing epitope are needed.

A series of serine-rich regions were found in GP4. More serine was observed in HP-like PRRSV when compared them with typical type II PRRSV. Serine makes the protein more flexible. This character may contribute to the easier combination of HP-PRRSV with the receptor. Thus, HP-PRRSV is more competitive when the two types of PRRSV simultaneously infect one cell. This finding may be the reason why HP-PRRSV has become increasingly prevalent in swine farms and why the less typical type PRRSV has been isolated from clinical samples [29]. This was also observed in our previous study when cells were co-infected with HP-PRRSV and typical PRRSV. Typical PRRSV cannot be detected after three passages on cells (unpublished data). Further research should focus on revealing the prelateship between serine-enriching in GP4 and virus infectivity.

## Conclusion

The GP4 antigen reaction panorama of HP-PRRSV created using the peptide scan method demonstrated the different reaction characteristics of the different regions in GP4. An antigen active reaction region that covered 24 aa from S29 to G56 was identified, and analysis of the region revealed the important function of the amino acids in site 43. The aa change in this site affects the antigenicity of HP-PRRSV GP4 when test with peptide based ELISA. Six epitope candidates were also identified in GP4, which may be used for identifying epitopes in further research.

## Additional files

Additional file 1: The peptides chip scanning results. (XLS 242 kb) Additional file 2: The peptide based ELLISA results. (XLSX 17 kb)
